# High-Sensitivity C-Reactive Protein, Its Change, and Cognitive Function: A National Population-Based Cohort Study

**DOI:** 10.3390/brainsci13040658

**Published:** 2023-04-13

**Authors:** Yechuang Wang, Jialin Fu, Fang Liang, Theresa M. Oniffrey, Kai Ding, Jing Zeng, Justin B. Moore, Xianwu Luo, Rui Li

**Affiliations:** 1 School of Public Health, Wuhan University, Wuhan 430071, China; 2 Chengdu High-Tech Zone Center for Diseases Control and Prevention, Chengdu 610000, China; 3 Department of Internal Medicine, Section of Gerontology and Geriatric Medicine, Atrium Health Wake Forest Baptist, Medical Center Boulevard, Winston-Salem, NC 27157, USA; 4 Department of Implementation Science, Division of Public Health Sciences, Wake Forest University School of Medicine, Winston-Salem, NC 27101, USA; 5 School of Nursing, Wuhan University, Wuhan 430071, China

**Keywords:** Hs-CRP, Hs-CRP change, cognitive decline, cognitive impairment

## Abstract

This study aimed to evaluate the associations of baseline high-sensitivity C-reactive protein (Hs-CRP) and its change with subsequent cognitive decline and cognitive impairment. Data for this study were obtained from the China Health and Retirement Longitudinal Study, a national community-based prospective cohort study. Hs-CRP level and cognitive function were measured repeatedly over a 7-year follow-up. Linear mixed models and cox proportional hazard models were used to evaluate the associations. The study comprised 7385 participants (50.67% women, mean age 59.08 ± 8.86 years) with baseline Hs-CRP ranging from 0.03 to 178.10 mg/L (median: 1.01 mg/L, IQR: 0.55–2.11 mg/L). During a median of 5.79 years follow-up, the highest quartile of the Hs-CRP group showed a faster rate of cognitive decline (−0.0053 SD/year, *p* = 0.006) and a higher risk of cognitive impairment (HR 1.0814, *p* = 0.044) than those in the lowest quartile. Individuals in the elevated group of Hs-CRP change had a significantly faster cognitive decline (−0.0070 SD/year, *p* = 0.016) compared with those in the stable group. In this study, significant longitudinal associations between baseline Hs-CRP, elevated Hs-CRP, and long-term cognitive deterioration were observed. Hs-CRP level could perhaps serve as a predictor for cognitive deterioration in middle-aged and older adults.

## 1. Introduction

With the increasing proportion of the older population, the prevalence of dementia is increasing rapidly, particularly in China [1]. A nationwide cross-sectional study completed in 2018 estimated that 6% and 15% of Chinese older adults have dementia and mild cognitive impairment, representing approximately 15 million and 38 million cases, respectively [2]. Dementia is one of the most common and serious diseases in the aging population and severely affects the quality of later life, and a large amount of disability and mortality as well as a heavy burden on families and society are caused by dementia [3]. The prevention of dementia is the priority since there are currently no effective treatments [1]. During the long preclinical phase of dementia, mild cognitive impairment and accelerated cognitive decline are considered the primary clinical characteristics [4]. Therefore, the identification of potential risk factors for mild cognitive impairment and accelerated cognitive decline is of great significance for dementia prevention.

It has been indicated that inflammatory mechanisms may contribute to cognitive decline, which eventually results in dementia [5,6]. C-reactive protein (CRP) is an acute-phase inflammatory molecule that has drawn interest as a potential biomarker of underlying cognitive health and dementia. However, inconsistent results have been reported. Several studies showed elevated CRP to be statistically significantly associated with incident cognitive impairment and dementia [6,7,8,9], with some evidence of faster cognitive decline [10,11,12,13], while other studies did not reach this conclusion [14,15,16,17,18,19]. For example, some studies observed a protective effect of elevated CRP on cognitive function [14,15,16]. Discrepancies may be partly explained by differences in the sizes of samples, the distribution of age, and the duration of follow-up. In addition, the associations of elevated circulatory CRP with cognitive function may be moderated by race [20,21,22]. To date, although two cross-sectional studies have explored the association between CRP and cognitive function [23,24], whether elevated CRP is associated with faster cognitive decline or a higher risk of cognitive impairment has not been thoroughly explored in large-scale cohort studies among Chinese adults. Furthermore, an important limitation of the existing studies is that all the studies measured circulatory CRP level at a single time point. Single assessments of CRP level may be unable to capture individuals’ long-term inflammatory exposures and fail to provide information regarding longitudinal change in inflammation and cognitive function [10].

The China Health and Retirement Longitudinal Study (CHARLS), with its national community-based population and multiple measurements on CRP level and cognitive function, provides an excellent opportunity to evaluate the association of circulatory CRP and its change with subsequent cognitive decline and cognitive impairment. Therefore, by using data from CHARLS, we aimed to evaluate the associations of baseline Hs-CRP and its change with subsequent cognitive decline and cognitive impairment in a large national cohort, with over 7000 middle-aged and older Chinese adults.

## 2. Methods

### 2.1. Population

As detailed elsewhere [25], CHARLS is a nationally representative sample of community-dwelling Chinese people aged 45 years or over that used stratified multi-stage probability random cluster sampling. The baseline survey consisted of 17,708 subjects from 150 counties of 28 of China’s provinces. Follow-up surveys were conducted every two or three years and a fraction of new participants was enrolled in every survey. All participants signed written inform consent in the baseline and follow-up surveys. The CHARLS study was approved by the Ethics Review Committee of Peking University.

**Figure 1:**
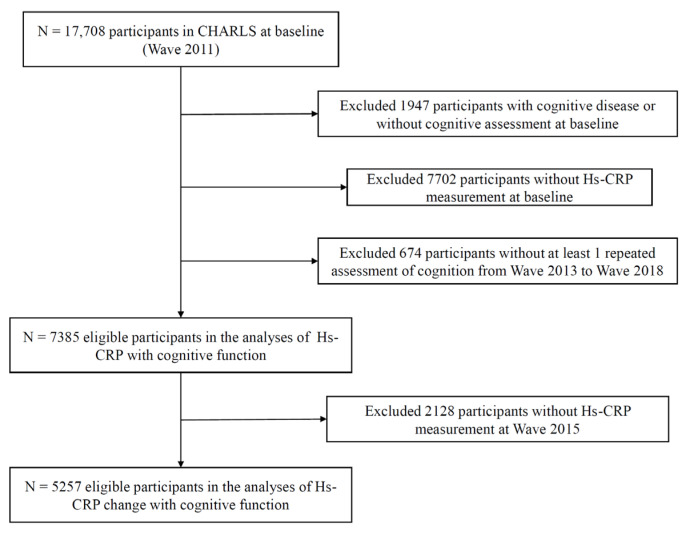
Flow chart of participants’ inclusion.

This study employed data from baseline (2011), Wave 2 (2013), Wave 3 (2015), and Wave 4 (2018) of the CHARLS. We excluded 1947 participants with cognitive disease or without cognitive assessment at baseline, and 7702 individuals due to missing Hs-CRP level at baseline. An additional 674 participants without at least 1 repeated assessment of cognition from Wave 2 to Wave 4 were excluded. We included 7385 participants in the analyses of the association of CRP with cognitive decline and cognitive impairment. Furthermore, we excluded 2128 participants without CRP measurement at Wave 3, resulting in a sample of 5257 participants enrolled in the analyses of the association of CRP change with cognitive decline and cognitive impairment. A flow chart of participant selection for the present study is shown in Figure 1.

### 2.2. Assessment of High-Sensitivity C-Reactive Protein

Venous blood samples were collected at baseline and follow-up survey, and blood-related indicators were measured. Hs-CRP was measured by immunoturbidimetry, which can provide more accurate measurement than standard CRP assays, particularly in normal healthy population [26]. Details of the process are available elsewhere [25,27].

In this study, following previous work [11], baseline Hs-CRP was categorized into quartiles as follows: quartile 1 (Hs-CRP < 0.55 mg/L), quartile 2 (0.55 mg/L ≤ Hs-CRP < 1.01 mg/L), quartile 3 (1.01 mg/L ≤ Hs-CRP < 2.11 mg/L), and quartile 4 (Hs-CRP ≥ 2.11 mg/L). Hs-CRP change was defined as second Hs-CRP level in 2015 (wave 3) minus first Hs-CRP level at baseline, and classified into 3 groups based on the results of the analyses of baseline CRP and cognitive function, including reduced group (Hs-CRP change < −2.11 mg/L), stable group (−2.11 mg/L≤ Hs-CRP change ≤ 2.11 mg/L), and elevated group (Hs-CRP change > 2.11 mg/L).

### 2.3. Assessment of Cognitive Function

Cognitive function was measured at baseline and every follow-up survey by various tests, including episodic memory, the Telephone Interview of Cognitive Status (TICS-10), and figure drawing. Respondents were asked to immediately repeat ten common nouns after the trained interviewers read the nouns to him/her, and they were again asked to recall the same nouns after four minutes. Episodic memory was computed as the sum of immediate and delayed recall (range 0–20). The items from TICS-10 included serial subtraction of 7 from 100 (up to 5 times), the date (day, month, year), day of the week, and season. Respondents were also requested to observe a picture of two overlapping pentagons and complete a similar figure (0 = unsuccessful completion; 1 = successful completion). Both the reliability and the validity of these tests have been well documented [28,29]. The global cognition score was created by summing the three tests scores, which has been widely used previously [30,31]. Higher score indicated better cognitive performance.

To enable comparison across cognitive tests, the standardized z scores for global cognition at each wave were computed according to the mean and standard deviation of the baseline scores. The rate of cognitive decline was calculated as the z score at follow-up minus the baseline z score and divided by the number of person-years of follow-up [32]. In addition, cognitive impairment was defined as the Chinese version of Mini-Mental State Examination (MMSE) score below 18 [33,34]. The Chinese MMSE is well documented to be both reliable and valid [35].

### 2.4. Assessment of Covariates

Covariates shown by prior research that may confound the association of Hs-CRP level with cognitive function were adjusted in our analyses. Potential confounders included age (in years), sex, type of residence (urban or rural), education (illiterate, primary school, or middle school and above), marital status (married or not), smoking status (current, former, or never), drinking status (current, former, or never), and sleep duration. In addition, depressive symptoms were evaluated by using the 10-item Center for Epidemiological Studies Depression Scale [36]. Chronic diseases were assessed via self-report. Each disease was given a point of 1 or 0 (presence vs absence) and the chronic diseases score ranged from 0 to 12. Height, weight, and systolic blood pressure (SBP) were objectively measured by trained interviewers with standardized equipment, and body mass index (BMI) was computed by the height and weight.

### 2.5. Statistical Analyses

The results are shown as mean and SD for normally distributed continuous variables or median and IQR for non-normally distributed continuous variables or by numbers and percentage for categorical variables.

Linear mixed models were developed to estimate the longitudinal association of baseline Hs-CRP and Hs-CRP change with consequent cognitive decline during follow-up using categories of baseline Hs-CRP and Hs-CRP change with the lowest group and the stable group as the reference, respectively. In the models, the intercept and slope were set as random effects, allowing for individual variations in baseline and the rates of change in cognitive function over time [37]. The first model included baseline Hs-CRP or Hs-CRP change, time (years since baseline), time × Hs-CRP or Hs-CRP change, age, sex, education, type of residence, and marital status. The second model additionally adjusted for baseline BMI, SBP, depressive symptoms, chronic diseases, smoking status, drinking status, and sleep duration. The third model further adjusted for baseline global cognitive score.

Cox proportional hazard models were then conducted to evaluate the association of baseline Hs-CRP and Hs-CRP change with cognitive impairment. Proportional hazards assumption was verified by using a global test for zero slope of the scaled Schoenfeld residuals over time. For both baseline Hs-CRP and Hs-CRP change, we calculated 3 models for the adjustment, including model 1, which included Hs-CRP or Hs-CRP change (the lowest group and the stable group as the reference, respectively), age, sex, education, type of residence, and marital status; model 2, additionally adjusted for baseline BMI, SBP, depressive symptoms, chronic diseases, smoking status, drinking status and sleep duration; model 3, further adjusted for baseline global cognitive score. In addition, we performed restricted cubic spline with knots placed at 10th, 50th, and 90th percentiles and used median value of baseline Hs-CRP (1.01 mg/L) and Hs-CRP change 0 mg/L as reference point to test the potential non-linear association between baseline Hs-CRP, its change, and cognitive impairment, respectively.

We imputed missing data for MMSE scores in 2018 (wave 4) by multiple imputation using chained equations, imputing 20 data sets [32]. Baseline information, including age, sex, education, type of residence and marital status, BMI, SBP, depressive symptoms, chronic diseases, smoking status, drinking status and sleep duration, and global cognitive score, were used to impute the missing data. All available MMSE scores data without multiple imputations in 2018 (wave 4) were used in the sensitivity analysis. In addition, since MMSE scores might be influenced by education level, we conducted another sensitivity analysis that applied education-specific cut-off points for defining cognitive impairment [38,39].

Data were analyzed using SAS 9.4 (SAS Institute, Cary, NC, USA) and R software, version 3.4.2 (R Foundation, Vienna, Austria). Tests were two-sided with statistical significance set as *p* < 0.05.

### 2.6. Data Availability

The data of this study are available to researchers on reasonable request by contacting the corresponding author.

## 3. Results

Of the 7385 participants included, 50.67% were females, and the mean (SD) age at baseline was 59.08 (8.86) years. The circulating Hs-CRP level across all participants ranged from 0.03 to 178.10 mg/L (median: 1.01 mg/L, IQR: 0.55–2.11 mg/L). Approximately 90.09% of the participants were married, 59.19% were never-smokers, 65.13% were never-drinkers, and 35.61% reported depressive symptoms at baseline. The BMI and SBP were 23.73 (3.93) kg/m^2^ and 130.71 (28.79) mmHg on average at baseline, respectively. The distribution of baseline covariates by baseline Hs-CRP level is shown in Table 1.

As listed in Table 2, the global cognitive scores of participants with the highest quartile of Hs-CRP decreased at a faster rate (−0.0055 SD/year, *p* = 0.011) than those in the lowest quartile in Model 1. The significance remained after accounting for covariates in Model 2 (−0.0052 SD/year, *p* = 0.026) and Model 3 (−0.0053 SD/year, *p* = 0.006).

We found that, after multivariable adjustment, participants in the elevated group had a significantly faster global cognitive score decline (−0.0070 SD/year, *p* = 0.016) compared with that of the stable group of Hs-CRP change (Table 3).

During a median of 5.79 years follow-up, a total of 1675 (22.68%) participants with incident cognitive impairment were identified. Table 4 shows the association of Hs-CRP with cognitive impairment. We found that, during a median of 5.79 years follow-up, compared with the lowest quartile, participants in the highest quartile showed a 1.0814-fold (95% CI 1.0020–1.1671) higher risk of cognitive impairment. The results were similar in the sensitivity analyses, which showed a 1.0954-fold (95% CI 1.0013–1.1984; Table S4) and a 1.1379-fold (95% CI 1.0141–1.2770; Table S5) higher risk, respectively.

Table 5 presents the association of Hs-CRP change with cognitive impairment. No significantly increased cognitive impairment risk was found in either the reduced group or the elevated group when compared to the stable group. The sensitivity analyses showed no substantial changes. (Tables S7 and S8).

**Figure 2:**
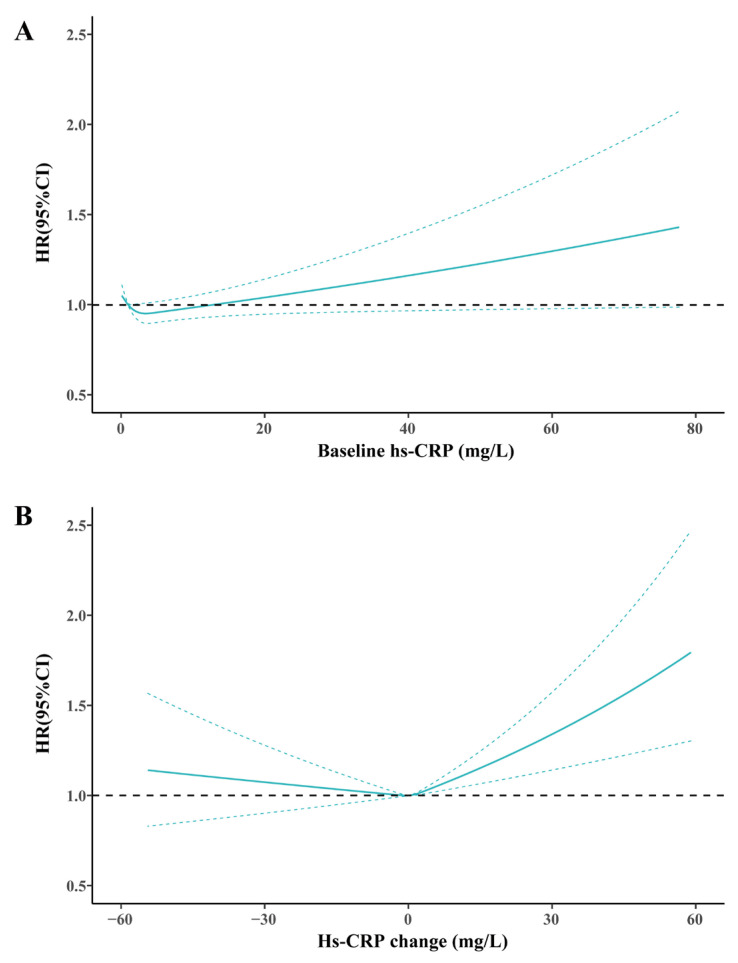
(**A**) Restricted cubic spline for the association of baseline Hs-CRP (as a continuous variable) with cognitive impairment. (**B**) Restricted cubic spline for the association of Hs-CRP change (as a continuous variable) with cognitive impairment. All covariates were age, sex, education, type of residence, marital status, BMI, SBP, depressive symptoms, chronic diseases, smoking status, drinking status, sleep duration, and global cognitive score. Reference point is 1.01 mg/L and 0 mg/L, respectively, with knots located at the 10th, 50th, and 90th percentiles. The solid lines represent multivariate-adjusted estimated hazard ratios and the dotted lines indicate the 95% CIs.

Baseline Hs-CRP was linearly associated with risk of cognitive impairment, with a positive and monotonic association (*p* for non-linear trend = 0.059). There was a non-linear association of Hs-CRP change with cognitive impairment (*p* for non-linear trend = 0.005), and the risk of cognitive impairment had a small decreasing trend until zero and then started to increase rapidly afterwards (Figure 2).

## 4. Discussion

To the best of our knowledge, this is the first study evaluating the association of Hs-CRP and its change with subsequent cognitive decline and cognitive impairment in a nationally representative cohort of Chinese adults. In the present study, we found participants in the highest quartile of Hs-CRP had a significantly faster cognitive decline and a significantly higher risk of cognitive impairment than those in the lowest quartile. In addition, individuals in the elevated group of Hs-CRP change had a significantly faster cognitive decline compared with those in the stable group.

The main finding of our study is that participants in the highest quartile of Hs-CRP had a significantly faster cognitive decline and a significantly higher risk of cognitive impairment over the 7 years of follow-up, which was consistent with several previous studies [8,10,11,12]. A large England cohort, conducted in middle-aged and older populations, demonstrated that individuals in the third and highest quartiles of Hs-CRP had a significantly faster cognitive decline than those in the lowest quartile [11]. It is worth noting that the range of the third quartiles’ Hs-CRP level group was 2.10 to 4.10 in the England cohort, and the range of the highest quartiles’ Hs-CRP level group was larger than 2.11 in our study. A recent US cohort reported that CRP was related to a faster rate of cognitive decline and a higher risk of dementia across a 10-year follow-up [10]. Similarly, a geriatric cross-sectional study from China showed increased CRP was associated with cognitive impairment [24]. Another cross-sectional study conducted in 3875 Chinese adults found that Hs-CRP was related to a significantly increased risk of cognitive impairment only among normal weight participants [23]. Future mechanism studies and epidemiological studies with larger sample sizes and a longer follow-up are required to determine the longitudinal association between Hs-CRP and cognitive change among Chinese adults.

The mechanisms that link CRP to cognitive deterioration are not completely understood, although several potential biological pathways have been identified. First, mounting evidence supports the role of inflammation in the development of cognitive dysfunction (e.g., dementia, delirium) [40]. CRP, as a representative acute-phase reactant, is regarded as an indicator of systemic inflammation [41].There is evidence that elevated CRP plays a crucial role in the development of cognitive dysfunction in both dementia and delirium [42,43]. The biological mechanism underlying the association of CRP with cognitive dysfunction can be, at least partially, explained by the inflammatory processes penetrating from the periphery into the central nervous system. Felger et al. suggested that peripheral CRP is closely related to its concentration in the cerebrospinal fluid, as well as other cytokines and their receptors, such as tumor necrosis factor alpha [44]. In addition, it is well-known that CRP enhances the permeability of the blood–brain barrier, thus allowing it to enter the brain directly [45]. Second, CRP has a strong correlation with structural brain changes, including alterations in total brain volume, gray matter and hippocampal volume, and white matter and microstructural integrity [46,47]. Third, CRP elevation has been related to alterations in metabolic and homeostatic systems, which have been associated with cognitive dysfunction [48].

We also found that individuals in the elevated group of Hs-CRP change had a significantly faster cognitive decline compared with those in the stable group. Emerging evidence demonstrates that elevated Hs-CRP may be an important marker of schizophrenia [49], cerebrovascular disease [50], and stroke [51]. However, to date, no studies have specifically explored the association of Hs-CRP variability with cognitive impairment or cognitive decline. We evaluated the association of long-term Hs-CRP change with subsequent cognitive decline and cognitive impairment utilizing a nationally representative cohort with a repeated measures design, and we found that elevated Hs-CRP is related to faster cognitive decline but not related to cognitive impairment. Future studies examining the association of CRP variability with cognitive function are needed.

A major strength of our study was the use of a high-quality, large-scale, nationally representative, prospective cohort of middle-aged and older Chinese adults, which enables more certainty in our findings. Moreover, we assessed cognitive function at multiple follow-up assessments, which is a reliable measure of cognitive deterioration, thus allowing us to reliably capture individual long-term trajectories of cognitive change. In addition, to date, this is the first research examining the association of Hs-CRP and its change with subsequent cognitive decline and cognitive impairment among a nationally representative cohort of Chinese adults. Moreover, we performed several sensitivity analyses with robust findings. This study has several limitations. The first is inherent to all observational studies in that we could detect only associations, not causality, from the observed associations. Secondly, large intervals between Hs-CRP measurements (four years) and relatively few assessments might limit the generalizability of the results. Moreover, in the present study, we classified Hs-CRP change into three groups according to the results of the analyses of baseline Hs-CRP and cognitive function; further studies are needed to validate our results. Finally, although we carefully adjusted for numerous potential confounders, we cannot rule out the potential of residual confounding.

In conclusion, based on the CHARLS cohort over a 7-year follow-up, we provide strong evidence for the correlation of Hs-CRP and its change with long-term cognitive deterioration. Our results indicate that Hs-CRP level could perhaps serve as a predictor of cognitive deterioration in the elderly. Our findings suggest more attention should be paid to Hs-CRP levels in older adults. Future research is required to determine the causality of this relationship and to elucidate the optimal Hs-CRP level for the prevention of cognitive deterioration in the elderly.

## Figures and Tables

**Table 1:** Baseline characteristics of study participants according to baseline Hs-CRP level.

	Total (*n* = 7385)	Quartile 1 (*n* = 1829)	Quartile 2 (*n* = 1873)	Quartile 3 (*n* = 1836)	Quartile 4 (*n* = 1847)
Age (years)	59.08 ± 8.86	57.20 ± 8.57	58.91 ± 8.87	59.72 ± 8.57	60.48 ± 9.08
Sex					
Male	3643 (49.33%)	844 (46.15%)	907 (48.42%)	931 (50.60%)	961 (52.14%)
Female	3742 (50.67%)	985 (53.85%)	966 (51.58%)	909 (49.40%)	882 (47.86%)
Education					
Illiterate	1507 (20.41%)	379 (20.72%)	378 (20.18%)	354 (19.24%)	396 (21.49%)
Primary school	3314 (44.87%)	815 (44.56%)	850 (45.38%)	835 (45.38%)	814 (44.17%)
Middle school and above	2564 (34.72%)	635 (34.72%)	645 (34.44%)	651 (35.38%)	633 (34.35%)
Marital status					
Married	6653 (90.09%)	1668 (91.20%)	1694 (90.44%)	1666 (90.54%)	1625 (88.17%)
Not married	732 (9.91%)	161 (8.80%)	179 (9.56%)	174 (9.46%)	218 (11.83%)
Smoking status					
Never smoking	4371 (59.19%)	1141 (62.38%)	1135 (60.60%)	1078 (58.59%)	1017 (55.18%)
Current smoking	2333 (31.59%)	561 (30.67%)	578 (30.86%)	568 (30.87%)	626 (33.97%)
Former smoking	681 (9.22%)	127 (6.94%)	160 (8.54%)	194 (10.54%)	200 (10.85%)
Drinking status					
Never drinking	4810 (65.13%)	1174 (64.19%)	1194 (63.75%)	1216 (66.09%)	1226 (66.52%)
Current drinking	1969 (26.66%)	494 (27.01%)	518 (27.66%)	464 (25.22%)	493 (26.75%)
Former drinking	606 (8.21%)	161 (8.80%)	161 (8.60%)	160 (8.70%)	124 (6.73%)
Depressive symptoms					
Yes	2630 (35.61%)	661 (36.14%)	681 (36.36%)	618 (33.59%)	670 (36.35%)
No	4755 (64.39%)	1168 (63.86%)	1192 (63.64%)	1222 (66.41%)	1173 (63.65%)
Chronic diseases scores	1 (0–1)	1 (0–1)	1 (0–1)	1 (0–2)	1 (0–2)
BMI (kg/m^2^)	23.73 ± 3.93	22.57 ± 3.33	23.49 ± 3.72	24.32 ± 3.88	24.53 ± 4.42
SBP (mmHg)	130.71 ± 28.79	126.4 ± 27.42	129.55 ± 27.11	132.23 ± 27.05	134.63 ± 32.56
Sleep duration (h)	6.40 ± 1.81	6.41 ± 1.82	6.42 ± 1.77	6.46 ± 1.82	6.30 ± 1.84
Hs-CRP (mg/L)	1.01 (0.55–2.11)	0.38 (0.30–0.46)	0.74 (0.64–0.85)	1.40 (1.19–1.71)	3.85 (2.73–6.68)
Global cognitive scores (2011)	15.16 ± 4.73	14.98 ± 4.88	15.12 ± 4.80	14.92 ± 4.84	15.61 ± 4.35
Global cognitive scores (2013)	13.56 ± 6.75	13.28 ± 6.63	13.41 ± 6.83	13.24 ± 6.85	14.29 ± 6.64
Global cognitive scores (2015)	12.30 ± 5.99	12.20 ± 6.07	12.18 ± 6.13	12.04 ± 6.07	12.78 ± 5.65
Global cognitive scores (2018)	10.76 ± 8.54	10.38 ± 8.56	10.46 ± 8.42	10.36 ± 8.53	11.84 ± 8.57

Data are mean (SD), median (IQR), or *n* (%); all participants were classified as quartile 1 (Hs-CRP < 0.55 mg/L), quartile 2 (0.55 mg/L ≤ Hs-CRP < 1.01 mg/L), quartile 3 (1.01 mg/L ≤ Hs-CRP < 2.11 mg/L), or quartile 4 (Hs-CRP ≥ 2.11 mg/L).

**Table 2:** Longitudinal analysis of mean difference in rate of change in global cognitive decline (SD per year) comparing quartiles of baseline high-sensitivity C-reactive protein (Hs-CRP).

	Quartile 1		Quartile 2		Quartile 3		Quartile 4	
	β (SE)	*p*-Value	β (SE)	*p*-Value	β (SE)	*p*-Value	β (SE)	*p*-Value
Model 1	0 (ref)		−0.0036 (0.0021)	0.090	0.0018 (0.0021)	0.404	−0.0055 (0.0022)	0.011
Model 2	0 (ref)		−0.0034 (0.0022)	0.114	0.0020 (0.0021)	0.344	−0.0052 (0.0023)	0.026
Model 3	0 (ref)		−0.0033 (0.0018)	0.065	0.0015 (0.0018)	0.395	−0.0053(0.0019)	0.006

Baseline Hs-CRP was classified as quartile 1 (Hs-CRP < 0.55 mg/L), quartile 2 (0.55 mg/L ≤ Hs-CRP < 1.01 mg/L), quartile 3 (1.01 mg/L ≤ Hs-CRP < 2.11 mg/L), or quartile 4 (Hs-CRP ≥ 2.11 mg/L); model 1: adjusted for baseline age, sex, education, type of residence, and marital status; model 2: further adjusted for baseline BMI, SBP, depressive symptoms, chronic diseases, smoking status, drinking status, and sleep duration; model 3: further adjusted for baseline global cognitive score. A detailed description is shown in Table S2.

**Table 3:** Longitudinal analysis of mean difference in rate of change in global cognitive decline (SD per year) comparing categories of high-sensitivity C-reactive protein (Hs-CRP) change.

	Reduced Group		Stable Group		Elevated Group	
	β (SE)	*p*-Value	β (SE)	*p*-Value	β (SE)	*p*-Value
Model 1	−0.0001 (0.0025)	0.955	0 (ref)		−0.0098 (0.0031)	0.002
Model 2	−0.0004 (0.0025)	0.880	0 (ref)		−0.0099 (0.0035)	0.004
Model 3	−0.0003 (0.0021)	0.990	0 (ref)		−0.0070 (0.0029)	0.016

Hs-CRP change was classified as reduced group (Hs-CRP change < −2.11 mg/L), stable group (−2.11 mg/L ≤ Hs-CRP change ≤ −2.11 mg/L), or elevated group (Hs-CRP change > 2.11 mg/L); model 1: adjusted for baseline age, sex, education, type of residence, and marital status; model 2: further adjusted for baseline BMI, SBP, depressive symptoms, chronic diseases, smoking status, drinking status, and sleep duration; model 3: further adjusted for baseline global cognitive score and Hs-CRP level. A detailed description is shown in Table S3.

**Table 4:** Association of baseline high-sensitivity C-reactive protein (Hs-CRP) with incident cognitive impairment risk.

	Quartile 1		Quartile 2		Quartile 3		Quartile 4	
	HR (95% CI)	*p*-Value	HR (95% CI)	*p*-Value	HR (95% CI)	*p*-Value	HR (95% CI)	*p*-Value
Model 1	1 (ref)		1.0387 (0.9646–1.1185)	0.315	0.9937 (0.9220–1.0710)	0.869	1.0860 (1.0085–1.1695)	0.029
Model 2	1 (ref)		1.0391 (0.9645–1.1194)	0.313	0.9929 (0.9198–1.0718)	0.855	1.0826 (1.0032–1.1684)	0.041
Model 3	1 (ref)		1.0389 (0.9643–1.1192)	0.315	0.9920 (0.9191–1.0708)	0.838	1.0814 (1.0020–1.1671)	0.044

Baseline Hs-CRP was classified as quartile 1 (Hs-CRP < 0.55 mg/L), quartile 2 (0.55 mg/L ≤ Hs-CRP < 1.01 mg/L), quartile 3 (1.01 mg/L ≤ Hs-CRP < 2.11 mg/L), or quartile 4 (Hs-CRP ≥ 2.11 mg/L); model 1: adjusted for baseline age, sex, education, type of residence, and marital status; model 2: further adjusted for baseline BMI, SBP, depressive symptoms, chronic diseases, smoking status, drinking status, and sleep duration; model 3: further adjusted for baseline global cognitive score. A detailed description is shown in Table S6.

**Table 5:** Association of high-sensitivity C-reactive protein (Hs-CRP) change with incident cognitive impairment risk.

	Reduced Group		Stable Group		Elevated Group	
	HR (95% CI)	*p*-Value	HR (95% CI)	*p*-Value	HR (95% CI)	*p*-Value
Model 1	0.9463 (0.8083–1.1078)	0.492	1 (ref)		0.8959 (0.7063–1.1364)	0.365
Model 2	0.9452 (0.8071–1.1068)	0.484	1 (ref)		0.8937 (0.7045–1.1338)	0.355
Model 3	0.9487 (0.8101–1.1111)	0.514	1 (ref)		0.8925 (0.7035–1.1323)	0.349

Hs-CRP change was classified as reduced group (Hs-CRP change < −2.11 mg/L), stable group (−2.11 mg/L ≤ Hs-CRP change ≤ 2.11 mg/L), or elevated group (Hs-CRP change > 2.11 mg/L); model 1: adjusted for baseline age, sex, education, type of residence, and marital status; model 2: further adjusted for baseline BMI, SBP, depressive symptoms, chronic diseases, smoking status, drinking status, and sleep duration; model 3: further adjusted for baseline global cognitive score and Hs-CRP level. A detailed description is shown in Table S9.

## Data Availability

The data of this study are available to researchers on reasonable request by contacting the corresponding author.

## Supplementary Materials

The following supporting information can be downloaded at: https://www.mdpi.com/article/10.3390/brainsci13040658/s1, Table S1: STROBE Statement—Checklist of items that should be included in reports of cohort studies; Table S2: Details of the longitudinal analysis of mean difference in rate of change in global cognitive decline (SD per year) comparing quartiles of baseline high-sensitivity C-reactive protein (Hs-CRP); Table S3: Details of the longitudinal analysis of mean difference in rate of change in global cognitive decline (SD per year) comparing categories of high-sensitivity C-reactive protein (Hs-CRP) change; Table S4: Sensitivity analysis for the association of baseline high-sensitivity C-reactive protein (Hs-CRP) with incident cognitive impairment risk, using education-specific cut-off points; Table S5: Sensitivity analysis for the association of baseline high-sensitivity C-reactive protein (Hs-CRP) with incident cognitive impairment risk, using all available data without multiple imputations; Table S6: Details of the longitudinal analysis of association of high-sensitivity C-reactive protein (Hs-CRP) change with incident cognitive impairment risk; Table S7: Sensitivity analysis for the association of high-sensitivity C-reactive protein (Hs-CRP) change with incident of cognitive impairment risk, using education-specific cut-off points; Table S8: Sensitivity analysis of association between high-sensitivity C-reactive protein (Hs-CRP) change and the incidence of cognitive impairment risk, using all available data without multiple imputations; Table S9: Details about the longitudinal analysis of association of high-sensitivity C-reactive protein (Hs-CRP) change with incident cognitive impairment risk.
