# Supplementary material for: High-Sensitivity C-Reactive Protein, Its Change, and Cognitive Function: A National Population-Based Cohort Study
Source: Brain Sci. 2023 Apr 13;13(4):658. doi: 10.3390/brainsci13040658 (PMC10137225; doi:10.3390/brainsci13040658)
Supplement: Supplementary file 1 [file brainsci-13-00658-s001.zip › brainsci-2240721-supplementary.pdf]

Supplementary Materials

Table S1. STROBE Statement—Checklist of items that should be included in reports of ***cohort studies***

	Item No	Recommendation	Page No
**Title and abstract**	1	(*a*) Indicate the study's design with a commonly used term in the title or the abstract	1
		(*b*) Provide in the abstract an informative and balanced summary of what was done and what was found	1
**Introduction**			
Background/rationale	2	Explain the scientific background and rationale for the investigation being reported	2
Objectives	3	State specific objectives, including any prespecified hypotheses	2
**Methods**			
Study design	4	Present key elements of study design early in the paper	3
Setting	5	Describe the setting, locations, and relevant dates, including periods of recruitment, exposure, follow-up, and data collection	3
Participants	6	(*a*) Give the eligibility criteria, and the sources and methods of selection of participants. Describe methods of follow-up	3
		(*b*) For matched studies, give matching criteria and number of exposed and unexposed	NA
Variables	7	Clearly define all outcomes, exposures, predictors, potential confounders, and effect modifiers. Give diagnostic criteria, if applicable	3-4
Data sources/ measurement	8*	For each variable of interest, give sources of data and details of methods of assessment (measurement). Describe comparability of assessment methods if there is more than one group	3-4
Bias	9	Describe any efforts to address potential sources of bias	3-4
Study size	10	Explain how the study size was arrived at	3
Quantitative variables	11	Explain how quantitative variables were handled in the analyses. If applicable, describe which groupings were chosen and why	3-5
Statistical methods	12	(*a*) Describe all statistical methods, including those used to control for confounding	5
		(*b*) Describe any methods used to examine subgroups and interactions	5-6
		(*c*) Explain how missing data were addressed	5-6
		(*d*) If applicable, explain how loss to follow-up was addressed	NA
		(*e*) Describe any sensitivity analyses	5-6
**Results**			
Participants	13*	(a) Report numbers of individuals at each stage of study—eg numbers potentially eligible, examined for eligibility, confirmed eligible, included in the study, completing follow-up, and analysed	3
		(b) Give reasons for non-participation at each stage	3
		(c) Consider use of a flow diagram	NA
Descriptive data	14*	(a) Give characteristics of study participants (eg demographic, clinical, social) and information on exposures and potential confounders	Table 1
		(b) Indicate number of participants with missing data for each variable of interest	3
		(c) Summarise follow-up time (eg, average and total amount)	1
Outcome data	15*	Report numbers of outcome events or summary measures over time	6-7

Main results	16	(*a*) Give unadjusted estimates and, if applicable, confounder-adjusted estimates and their precision (eg, 95% confidence interval). Make clear which confounders were adjusted for and why they were included	6-7
		(*b*) Report category boundaries when continuous variables were categorized	6-7
		(*c*) If relevant, consider translating estimates of relative risk into absolute risk for a meaningful time period	NA
Other analyses	17	Report other analyses done—eg analyses of subgroups and interactions, and sensitivity analyses	6-7
**Discussion**			
Key results	18	Summarise key results with reference to study objectives	7-8
Limitations	19	Discuss limitations of the study, taking into account sources of potential bias or imprecision. Discuss both direction and magnitude of any potential bias	8-9
Interpretation	20	Give a cautious overall interpretation of results considering objectives, limitations, multiplicity of analyses, results from similar studies, and other relevant evidence	9
Generalisability	21	Discuss the generalisability (external validity) of the study results	9
**Other information**			
Funding	22	Give the source of funding and the role of the funders for the present study and, if applicable, for the original study on which the present article is based	NA

*Give information separately for exposed and unexposed groups.

Note: An Explanation and Elaboration article discusses each checklist item and gives methodological background and published examples of transparent reporting. The STROBE checklist is best used in conjunction with this article (freely available on the Web sites of PLoS Medicine at http://www.plosmedicine.org/, Annals of Internal Medicine at http://www.annals.org/, and Epidemiology at http://www.epidem.com/). Information on the STROBE Initiative is available at http://www.strobe-statement.org.

Table S2. Details of the longitudinal analysis of mean difference in rate of change in global cognitive decline (SD per year) comparing quartiles of baseline high-sensitivity C-reactive protein (Hs-CRP).

	**Model 1**		**Model 2**		**Model 3**	
	**β (SE)**	***p* value**	**β (SE)**	***p* value**	**β (SE)**	***p* value**
Baseline Hs-CRP						
Quartile 1	0 (ref)		0 (ref)		0 (ref)	
Quartile 2	-0.0036 (0.0021)	0.090	-0.0034 (0.0022)	0.114	-0.0033 (0.0018)	0.065
Quartile 3	0.0018 (0.0021)	0.404	0.0020 (0.0021)	0.344	0.0015 (0.0018)	0.395
Quartile 4	-0.0055 (0.0022)	0.011	-0.0052 (0.0023)	0.026	-0.0053 (0.0019)	0.006
Age	-0.0004 (0.0005)	0.446	-0.0001 (0.0005)	0.239	-0.0027 (0.0004)	< 0.001
Sex						
Male	-0.0018 (0.0079)	0.823	0.0057 (0.0092)	0.537	0.0074 (0.0083)	0.368
Female	0 (ref)		0 (ref)		0 (ref)	
Education						
Illiterate	-0.0212 (0.0066)	0.001	-0.0239 (0.0067)	< 0.001	-0.1498 (0.0058)	< 0.001
Primary school	-0.0041 (0.0049)	0.407	-0.0056 (0.0050)	0.260	-0.0620 (0.0040)	< 0.001
Middle school and above	0 (ref)		0 (ref)		0 (ref)	
Type of residence						
urban	-0.0068 (0.0077)	0.376	-0.0068 (0.0078)	0.383	-0.0518 (0.0061)	< 0.001
rural	0 (ref)		0 (ref)		0 (ref)	
Marital status						
Married	0.0157 (0.0074)	0.033	0.0183 (0.0075)	0.015	0.0233 (0.0059)	< 0.001
Not married	0 (ref)		0 (ref)		0 (ref)	
BMI (kg/m^2)			-0.0014 (0.0006)	0.019	0.0001 (0.0001)	0.035
SBP (mmHg)			-0.0001 (0.0001)	0.577	0.0010 (0.0005)	0.037
Depressive symptoms						
Yes			-0.0174 (0.0047)	< 0.001	-0.0213 (0.0037)	< 0.001
No			0 (ref)		0 (ref)	
Chronic diseases scores			0.0097 (0.0023)	< 0.001	0.0078 (0.0018)	< 0.001
Smoking status						
Never smoking			0.0187 (0.0063)	0.003	0.0158 (0.0049)	0.001
Current smoking			-0.0005 (0.0080)	0.953	0.0079 (0.0061)	0.201
Former smoking			0 (ref)		0 (ref)	
Drinking status						
Never drinking			-0.0168 (0.0056)	0.003	-0.008 (0.0043)	0.059
Current drinking			-0.0062 (0.0084)	0.463	0.0052 (0.0065)	0.428
Former drinking			0 (ref)		0 (ref)	
Sleep duration (h)			-0.0005 (0.0012)	0.692	0.0013 (0.0009)	0.156
Baseline global cognitive score					-0.0041 (0.0019)	0.028

Baseline Hs-CRP was classified as quartile 1 (Hs-CRP < 0.55 mg/L), quartile 2 (0.55 mg/L ≤ Hs-CRP < 1.01 mg/L), quartile 3 (1.01 mg/L ≤ Hs-CRP < 2.11 mg/L), and quartile 4 (Hs-CRP ≥ 2.11 mg/L).

Table S3. Details of the longitudinal analysis of mean difference in rate of change in global cognitive decline (SD per year) comparing categories of high-sensitivity C-reactive protein (Hs-CRP) change.

	**Model 1**		**Model 2**		**Model 3**	
	**β (SE)**	***p* value**	**β (SE)**	***p* value**	**β (SE)**	***p* value**
Hs-CRP change						
Reduced Group	-0.0001 (0.0025)	0.955	-0.0004 (0.0025)	0.880	-0.0003 (0.0021)	0.990
Stable Group	0 (ref)		0 (ref)		0 (ref)	
Elevated Group	-0.0098 (0.0031)	0.002	-0.0099 (0.0035)	0.004	-0.0070 (0.0029)	0.016
Age	-0.0001 (0.0005)	0.803	-0.0004 (0.0006)	0.523	-0.0026 (0.0005)	< 0.001
Sex						
Male	-0.0049 (0.0093)	0.604	0.0035 (0.0109)	0.745	0.0147 (0.0097)	0.132
Female	0 (ref)		0 (ref)		0 (ref)	
Education						
Illiterate	-0.0356 (0.0078)	< 0.001	-0.0377 (0.0080)	< 0.001	-0.1694 (0.0066)	< 0.001
Primary school	-0.0128 (0.0059)	0.029	-0.0135 (0.0059)	0.023	-0.0675 (0.0047)	< 0.001
Middle school and above	0 (ref)		0 (ref)		0 (ref)	
Type of residence						
urban	0.0009 (0.0098)	0.923	0.0023 (0.0099)	0.819	-0.0454 (0.0076)	< 0.001
rural	0 (ref)		0 (ref)		0 (ref)	
Marital status						
Married	0.0251 (0.0088)	0.005	0.0271 (0.0089)	0.015	0.0288 (0.0070)	< 0.001
Not married	0 (ref)		0 (ref)		0 (ref)	
BMI (kg/m^2)			-0.0010 (0.0007)	0.141	0.0010 (0.0005)	0.005
SBP (mmHg)			-0.0001 (0.0001)	0.571	-0.0002 (0.0006)	0.030
Depressive symptoms						
Yes			-0.0121 (0.0056)	0.031	-0.0239 (0.0044)	< 0.001
No			0 (ref)		0 (ref)	
Chronic diseases scores			0.0110 (0.0027)	< 0.001	0.0084 (0.0021)	< 0.001
Smoking status						
Never smoking			0.0208 (0.0074)	0.005	0.0191 (0.0057)	< 0.001
Current smoking			0.0020 (0.0094)	0.828	0.0135 (0.0073)	0.064
Former smoking			0 (ref)		0 (ref)	
Drinking status						
Never drinking			-0.0155 (0.0066)	0.019	-0.0074 (0.0051)	0.150
Current drinking			-0.0007 (0.0099)	0.938	0.0089 (0.0076)	0.242
Former drinking			0 (ref)		0 (ref)	
Sleep duration (h)			-0.0002 (0.0014)	0.872	0.0013 (0.0011)	0.252
Baseline global cognitive score					-0.1343 (0.0023)	< 0.001

Hs-CRP change was classified as reduced group (Hs-CRP change < -2.11 mg/L), stable group (-2.11 mg/L ≤ Hs-CRP change ≤ -2.11 mg/L), and elevated group (Hs-CRP change > 2.11 mg/L).

Table S4. Sensitivity analysis for the association of baseline high-sensitivity C-reactive protein (Hs-CRP) with incident cognitive impairment risk, using education-specific cut-off points.

	Quartile 1		Quartile 2		Quartile 3		Quartile 4	
	**HR (95 % CI)**	***p* value**	**HR (95 % CI)**	***p* value**	**HR (95 % CI)**	***p* value**	**HR (95 % CI)**	***p* value**
Model 1	1 (ref)		1.0226 (0.9364 – 1.1167)	0.619	0.9724 (0.8894 – 1.0632)	0.539	1.1007 (1.0087 – 1.2012)	0.031
Model 2	1 (ref)		1.022 (0.9354 – 1.1167)	0.630	0.9705 (0.8862 – 1.0629)	0.519	1.0958 (1.0017 – 1.1988)	0.046
Model 3	1 (ref)		1.0208 (0.9342 – 1.1153)	0.649	0.9700 (0.8857 – 1.0623)	0.512	1.0954 (1.0013 – 1.1984)	0.047

Baseline Hs-CRP was classified as quartile 1 (Hs-CRP < 0.55 mg/L), quartile 2 (0.55 mg/L ≤ Hs-CRP < 1.01 mg/L), quartile 3 (1.01 mg/L ≤ Hs-CRP < 2.11 mg/L), and quartile 4 (Hs-CRP ≥ 2.11 mg/L); model 1: adjusted for baseline age, sex, education, type of residence and marital status; model 2: further adjusted for baseline BMI, SBP, depressive symptoms, chronic diseases, smoking status, drinking status and sleep duration; model 3: further adjusted for baseline global cognitive score.

Table S5. Sensitivity analysis for the association of baseline high-sensitivity C-reactive protein (Hs-CRP) with incident cognitive impairment risk, using all available data without multiple imputations.

	Quartile 1		Quartile 2		Quartile 3		Quartile 4	
	**HR (95 % CI)**	***p* value**	**HR (95 % CI)**	***p* value**	**HR (95 % CI)**	***p* value**	**HR (95 % CI)**	***p* value**
Model 1	1 (ref)		1.0798 (0.9667 – 1.1206)	0.174	1.0282 (0.9192 – 1.1501)	0.627	1.1481 (1.0278 – 1.2825)	0.014
Model 2	1 (ref)		1.0819 (0.9671 – 1.2104)	0.169	1.0264 (0.9149 – 1.1516)	0.657	1.1381 (1.0141 – 1.2771)	0.028
Model 3	1 (ref)		1.0816 (0.9667 – 1.2102)	0.171	1.0263 (0.9148 – 1.1515)	0.658	1.1379 (1.0141 – 1.2770)	0.028

Baseline Hs-CRP was classified as quartile 1 (Hs-CRP < 0.55 mg/L), quartile 2 (0.55 mg/L ≤ Hs-CRP < 1.01 mg/L), quartile 3 (1.01 mg/L ≤ Hs-CRP < 2.11 mg/L), and quartile 4 (Hs-CRP ≥ 2.11 mg/L); model 1: adjusted for baseline age, sex, education, type of residence and marital status; model 2: further adjusted for baseline BMI, SBP, depressive symptoms, chronic diseases, smoking status, drinking status and sleep duration; model 3: further adjusted for baseline global cognitive score.

Table S6. Details of the longitudinal analysis of association of high-sensitivity C-reactive protein (Hs-CRP) change with incident cognitive impairment risk.

	Model 1		Model 2		Model 3	
	**HR (95 % CI)**	***p* value**	**HR (95 % CI)**	***p* value**	**HR (95 % CI)**	***p* value**
Baseline Hs-CRP						
Quartile 1	1 (ref)		1 (ref)		1 (ref)	
Quartile 2	1.0387 (0.9646 – 1.1185)	0.315	1.0391 (0.9645 – 1.1194)	0.313	1.0389 (0.9643 – 1.1192)	0.315
Quartile 3	0.9937 (0.9220 – 1.0710)	0.869	0.9929 (0.9198 – 1.0718)	0.855	0.9920 (0.9191 – 1.0708)	0.838
Quartile 4	1.0860 (1.0085 – 1.1695)	0.029	1.0826 (1.0032 – 1.1684)	0.041	1.0814 (1.0020 – 1.1671)	0.044
Age	1.0119 (1.0086 – 1.0153)	< 0.001	1.0114 (1.0078 – 1.0150)	< 0.001	1.0102 (1.0066 – 1.0137)	< 0.001
Sex						
Male	0.9893 (0.9360 – 1.0457)	0.703	1.0178 (0.9419 – 1.0998)	0.655	1.0241 (0.9977 – 1.1066)	0.547
Female	1 (ref)		1 (ref)		1 (ref)	
Education						
Illiterate	1.4172 (1.3071 – 1.5366)	< 0.001	1.4082 (1.2982– 1.5275)	< 0.001	1.2716 (1.1656 – 1.3873)	< 0.001
Primary school	1.0833 (1.0186 – 1.1521)	0.011	1.0763 (1.0116– 1.1451)	0.020	1.0322 (0.9689 – 1.0996)	0.326
Middle school and above	1 (ref)		1 (ref)		1 (ref)	
Type of residence						
urban	0.9393 (0.8551 – 1.0317)	0.191	0.9359 (0.8517 – 1.0286)	0.169	0.8989 (0.8173 – 0.9887)	0.028
rural	1 (ref)		1 (ref)		1 (ref)	
Marital status						
Married	0.9646 (0.8802 – 1.0571)	0.441	0.9707 (0.8854 – 1.0642)	0.526	0.9714 (0.8860 – 1.0650)	0.536
Not married	1 (ref)		1 (ref)		1 (ref)	
BMI (kg/m^2)			0.9993 (0.9919 – 1.0068)	0.857	1.0014 (0.9940 – 1.0089)	0.709
SBP (mmHg)			1.0002 (0.9993 – 1.0011)	0.667	1.0001 (0.9992 – 1.0010)	0.775
Depressive symptoms						
Yes			1.0460 (0.9873 – 1.1081)	0.127	1.0152 (0.9576 – 1.0762)	0.613
No			1 (ref)		1 (ref)	
Chronic diseases scores			1.0054 (0.9781 – 1.0335)	0.702	1.0039 (0.9765 – 1.0320)	0.784
Smoking status						
Never smoking			0.9875 (0.8899 – 1.0960)	0.813	0.9943 (0.8961 – 1.1034)	0.915
Current smoking			0.9977 (0.9241 – 1.0771)	0.953	0.9977 (0.9240 – 1.0771)	0.952
Former smoking			1 (ref)		1 (ref)	
Drinking status						

Never drinking	0.9428 (0.8530 – 1.0419)	0.248	0.9428 (0.8531 – 1.0420)	0.249
Current drinking	0.9610 (0.8972 – 1.0295)	0.257	0.9539 (0.8905 – 1.0218)	0.179
Former drinking	1 (ref)		1 (ref)	
Sleep duration (h)	0.9967 (0.9818– 1.0117)	0.662	0.9983 (0.9834 – 1.0133)	0.820
Baseline global cognitive score			0.9029 (0.8754 – 0.9312)	< 0.001

Baseline Hs-CRP was classified as quartile 1 (Hs-CRP < 0.55 mg/L), quartile 2 (0.55 mg/L ≤ Hs-CRP < 1.01 mg/L), quartile 3 (1.01 mg/L ≤ Hs-CRP < 2.11 mg/L), and quartile 4 (Hs-CRP ≥ 2.11 mg/L).

Table S7. Sensitivity analysis for the association of high-sensitivity C-reactive protein (Hs-CRP) change with incident of cognitive impairment risk, using education-specific cut-off points.

	**Reduced group**		**Stable group**		**Elevated group**	
	**HR (95 % CI)**	***p* value**	**HR (95 % CI)**	***p* value**	**HR (95 % CI)**	***p* value**
Model 1	0.9116 (0.8152 – 1.0194)	0.105	1 (ref)		0.8963 (0.7582 – 1.0595)	0.199
Model 2	0.9095 (0.8132 – 1.0172)	0.097	1 (ref)		0.8973 (0.7590 – 1.0609)	0.205
Model 3	0.9115 (0.8149 – 1.0195)	0.105	1 (ref)		0.8964 (0.7582 – 1.0598)	0.201

Hs-CRP change was classified as including reduced group (Hs-CRP change < -2.11 mg/L), stable group (-2.11 mg/L ≤ Hs-CRP change ≤ 2.11 mg/L), and elevated group (Hs-CRP change > 2.11 mg/L); model 1: adjusted for baseline age, sex, education, type of residence and marital status; model 2: further adjusted for baseline BMI, SBP, depressive symptoms, chronic diseases, smoking status, drinking status and sleep duration; model 3: further adjusted for baseline global cognitive score and Hs-CRP level.

Table S8. Sensitivity analysis of association between high-sensitivity C-reactive protein (Hs-CRP) change and the incidence of cognitive impairment risk, using all available data without multiple imputations.

	Reduced group		Stable group		Elevated group	
	**HR (95 % CI)**	***p* value**	**HR (95 % CI)**	***p* value**	**HR (95 % CI)**	***p* value**
Model 1	0.8761 (0.7022 – 1.0931)	0.241	1 (ref)		0.9631 (0.6937 – 1.3371)	0.822
Model 2	0.8625 (0.6903 – 1.0778)	0.193	1 (ref)		0.9290 (0.6659 – 1.2960)	0.665
Model 3	0.8624 (0.6902 – 1.0776)	0.193	1 (ref)		0.9300 (0.6665 – 1.2975)	0.669

Hs-CRP change was classified as including reduced group (Hs-CRP change < -2.11 mg/L), stable group (-2.11 mg/L ≤ Hs-CRP change ≤ 2.11 mg/L), and elevated group (Hs-CRP change > 2.11 mg/L); model 1: adjusted for baseline age, sex, education, type of residence and marital status; model 2: further adjusted for baseline BMI, SBP, depressive symptoms, chronic diseases, smoking status, drinking status and sleep duration; model 3: further adjusted for baseline global cognitive score and Hs-CRP level.

Table S9. Details about the longitudinal analysis of association of high-sensitivity C-reactive protein (Hs-CRP) change with incident cognitive impairment risk.

	Model 1		Model 2		Model 3	
	**HR (95 % CI)**	***p* value**	**HR (95 % CI)**	***p* value**	**HR (95 % CI)**	***p* value**
Hs-CRP change						
Reduced group	0.9463 (0.8083 – 1.1078)	0.492	0.9452 (0.8071 – 1.1068)	0.484	0.9487 (0.8101 – 1.1111)	0.514
Stable group	1 (ref)		1 (ref)		1 (ref)	
Elevated group	0.8959 (0.7063 – 1.1364)	0.365	0.8937 (0.7045 – 1.1338)	0.355	0.8925 (0.7035 – 1.1323)	0.349
Age	0.9978 (0.9905 – 1.0051)	0.552	0.9975 (0.9899 – 1.0051)	0.515	0.9970 (0.9894 – 1.0046)	0.435
Sex						
Male	0.9443 (0.8368 – 1.0656)	0.353	1.0491 (0.8816 – 1.2483)	0.589	1.0464 (0.8794 – 1.2452)	0.609
Female	1 (ref)		1 (ref)		1 (ref)	
Education						
Illiterate	0.6942 (0.5942 – 0.8110)	< 0.001	0.7100 (0.6074– 0.8299)	< 0.001	0.7283 (0.6197 – 0.8558)	< 0.001
Primary school	0.5989 (0.5029 – 0.7133)	< 0.001	0.6093 (0.5110– 0.7266)	< 0.001	0.6363 (0.5264 – 0.7692)	< 0.001
Middle school and above	1 (ref)		1 (ref)		1 (ref)	
Type of residence						
urban	0.8714 (0.6989 – 1.0865)	0.221	0.8879 (0.7115 – 1.1081)	0.293	0.9015 (0.7214 – 1.1266)	0.362
rural	1 (ref)		1 (ref)		1 (ref)	
Marital status						
Married	1.3684 (1.1329 – 1.6530)	< 0.001	1.3567 (1.1215 – 1.6412)	0.002	1.3565 (1.1213 – 1.6411)	0.002
Not married	1 (ref)		1 (ref)		1 (ref)	
BMI (kg/m^2)			0.9842 (0.9680 – 1.0008)	0.062	0.9851 (0.9688 – 1.0018)	0.079
SBP (mmHg)			1.0019 (1.0002 – 1.0037)	0.018	1.0020 (1.0002 – 1.0004)	0.028
Depressive symptoms						
Yes			0.9260 (0.8141 – 1.0533)	0.242	0.9141 (0.8023 – 1.0415)	0.177
No			1 (ref)		1 (ref)	
Chronic diseases scores			0.9263 (0.8692 – 0.9872)	0.018	0.9254 (0.8683 – 0.9864)	0.017
Smoking status						
Never smoking			1.0842 (0.8658 – 1.3578)	0.481	1.0855 (0.8669 – 1.3592)	0.475
Current smoking			1.0260 (0.8647 – 1.2175)	0.768	1.0256 (0.8643– 1.2170)	0.772
Former smoking			1 (ref)		1 (ref)	
Drinking status						
Never drinking			1.0844 (0.8838 – 1.3304)	0.438	1.0848 (0.884 – 1.3310)	0.436

Current drinking	1.0827 (0.9323 – 1.2573)	0.298	1.0801 (0.9300 – 1.2545)	0.313
Former drinking	1 (ref)		1 (ref)	
Sleep duration (h)	0.9965 (0.9637– 1.0305)	0.768	0.9973 (0.9644 – 1.0312)	0.873
Baseline global cognitive score			0.9586 (0.8951 – 1.0266)	0.226

Hs-CRP change was classified as reduced group (Hs-CRP change < -2.11 mg/L), stable group (-2.11 mg/L ≤ Hs-CRP change ≤ -2.11 mg/L), and elevated group (Hs-CRP change > 2.11 mg/L).
